# Recovery from Different High-Intensity Interval Training Protocols: Comparing Well-Trained Women and Men

**DOI:** 10.3390/sports9030034

**Published:** 2021-03-02

**Authors:** Laura Hottenrott, Martin Möhle, Alexander Ide, Sascha Ketelhut, Oliver Stoll, Kuno Hottenrott

**Affiliations:** 1 Institute of Performance Diagnostics and Health Promotion, Martin-Luther-University Halle-Wittenberg, 06108 Halle, Germany; oliver.stoll@sport.uni-halle.de (O.S.); kuno.hottenrott@sport.uni-halle.de (K.H.); 2 Institute of Sport Science, Martin-Luther University Halle-Wittenberg, 06108 Halle, Germany; martin.moehle@sport.uni-halle.de (M.M.); alexander.ide@sport.uni-halle.de (A.I.); sascha.ketelhut@sport.uni-halle.de (S.K.)

**Keywords:** wingate test, HIIT, interval training, sex differences, female athletes, cycling, endurance exercise, lactate, heart rate recovery, perceived exertion

## Abstract

Due to physiological and anatomical sex differences, there are variations in the training response, and the recovery periods following exercise may be different. High-intensity interval training (HIIT) protocols are well-suited to differentially investigate the course of recovery. This study was conducted to determine sex-specific differences in the recovery following HIIT intervals interspersed with recovery phases of different lengths. Methods: Well-trained cyclists and triathletes (n = 11 females, n = 11 males) participated in this study. There were no significant sex differences in maximal heart rate (HR), relative peak power to body mass and fat-free mass, training volume, and VO_2max_-percentiles (females: 91.8 ± 5.5 %, males: 94.6 ± 5.4 %). A 30 s Wingate test was performed four times, separated by different active recovery periods (1, 3, or 10 min). Lactate, HR, oxygen uptake, and subjective rating of exertion and recovery were determined. Results: For the recovery time of three and ten minutes, men showed significantly higher lactate concentrations (*p* = 0.04, *p* = 0.004). Contrary, HR recovery and subjective recovery were significant slower in women than in men. Conclusion: During HIIT, women may be more resistant to fatigue and have a greater ability to recover metabolically, but have a slower HR and subjective recovery.

## 1. Introduction

High-intensity interval training (HIIT) sessions are increasingly being used in performance and recreational sports to improve endurance performance and maximum oxygen uptake (VO_2max_) [1,2,3]. HIIT protocols are infinitely variable and may differ in terms of intensity, duration of intervals, number of repetitions and recovery time between interval bouts, thereby pursuing different training goals [4,5]. Postexercise recovery, as a multifaceted (e.g., physiological, psychological) restorative process, is a fundamental component of exercise training and is crucial for continuous performance development [6,7]. The duration of the recovery time influences the maximal performance during each interval and the overall organismic stress [8,9]. Furthermore, HIIT protocols are well-suited for differentially investigating the course of recovery in women and men.

Previous research has shown that due to physiological and anatomical sex differences, there are variations in the training response between women and men, and the recovery period following exercise may be characterized by different processes [10]. Sprint performance during intermittent exercise on a cycle ergometer is on average higher in men than in women [11]. On the other hand, women appear to have a higher resistance to fatigue [12,13]. Regarding intermittent exercise, this might require sex-specific recovery times during and after exercise to achieve the intended training effects. Currently, there are no specific recommendations that differentially address the recovery of endurance-trained women and men during and after intensive intermittent endurance exercise. During isometric contractions, Albert et al. [14] were able to show sex-specific differences in the recovery process, whereby women demonstrated a higher fatigue resistance and a higher relative performance than men. This was also confirmed by Wüst et al. [15] for isometric exercises.

Sex-specific differences in the recovery process during repeated cycling ergometer sprints over 30 s (Wingate tests with 20 min rest in between) have been revealed by Esbjörnsson-Liljedahl et al. [16]. They reported a faster Adenosine triphosphate ATP resynthesis in muscle biopsies during the recovery phases in women. Accordingly, there is some evidence that the course of recovery may differ between men and women during and after high levels of physical exertion. However, how sex affects the recovery has not been assessed during HIIT protocols applying different rest periods between the exercise bouts.

Maximal efforts over 30 s on the cycle ergometer (Wingate anaerobic test (WAnT)) are well-suited for the obtention of standardized power and recovery data [17] and to allow for continuous measurement of power, heart rate, and oxygen uptake. The duration of 30 s has been proven to be suitable to evaluate anaerobic performance in numerous experimental studies on maximal exercise [18]. It also allows for a comprehensive and differentiated discussion of the results of this study with previous results from WAnTs and for the derivation of practical conclusions (e.g., for the control of interval training sessions in different endurance sports). So far, there have been no studies assessing sex differences in recovery after different HIIT protocols. Thus, investigating repeated 30-s HIIT periods to examine sex differences in recovery variables addresses a gap in the literature.

This study aims to examine whether there are sex-specific differences in metabolic, cardiovascular, and subjective recovery following 30-s high-intensity intervals interspersed with recovery phases of different lengths. Furthermore, it will be assessed whether the different recovery periods influence the maximal power output of female and male athletes.

## 2. Materials and Methods

### 2.1. Participants

Twenty-four well-trained endurance athletes (cyclists and triathletes), including 12 females (mean age: 32.1 ± 9.7 years) and 12 males (mean age: 33.2 ± 9.9 years), were recruited to take part in this study. Two athletes (one male, one female) dropped out due to injuries that were not related to the study intervention. Participation required a training volume of at least 6 h/week of endurance exercise and cycling training for at least six months prior to the intervention. Additionally, athletes had to meet the following inclusion criteria: very good endurance performance (above the 80th percentile) based on VO_2max_ [19]. The baseline characteristics of the 22 athletes included in the data analysis are shown in Table 1. There were no significant differences between women and men concerning the performance-related parameters maximal heart rate (HR_max_), relative peak power output to body mass and fat-free mass (FFM), and weekly training volume. According to age and sex-specific VO_2max_ percentiles [19], female and male athletes displayed a comparable maximal aerobic performance capacity. Analysis of the bioimpedance data showed sex differences for body mass, body mass index (BMI), body fat, and FFM. There were no significant changes in bioimpedance data between testing days. This study was carried out in accordance with the Declaration of Helsinki and approved by the Martin Luther University Halle Wittenberg Ethics Committee (Reference code: 2019-094). All participants were informed about the risks and benefits of the investigation and provided informed consent before participating in the study.

### 2.2. Test Protocol

Participants reported to the laboratory on four occasions in a rested and hydrated state after fasting for at least two hours. Furthermore, they were told to avoid strenuous exercise 48 h before all tests. Each athlete was examined at the same time of day, and performance tests were conducted on the same cycling ergometer. Throughout the course of the study, participants were asked to maintain their usual dietary habits and training load was documented. All tests were conducted under standardized conditions (20 °C room temperature and 55% relative humidity).

During the first visit, baseline assessments were conducted. Participants completed a medical questionnaire to ensure they were not taking any medication or supplements that could interfere with the study. Additionally, body composition (body mass, body fat, and FFM) was measured using a bioimpedance device (Bio Impedance Analyzer, Data Input GmbH, Germany) after 20 min supine rest. Afterwards, aerobic fitness in terms of oxygen uptake was assessed using a Metalyzer 3B (Cortex, Leipzig, Germany) during an incremental test until voluntary exhaustion on a high-performance bicycle ergometer (E 2000 s, FES, Berlin, Germany). The test started with an eight-minute warm-up on the cycling ergometer at 100 (male athletes) or 70 W (female athletes). After the warm-up phase, the athletes completed the VO_2max_ test. All participants started with a resistance of 70 W. Every minute, the power was increased by 30 W. The cadence was set at 80–90 rpm.

**Figure 1:**
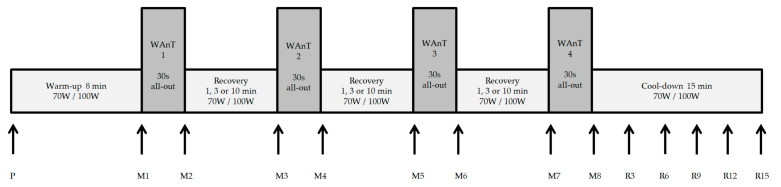
Study procedure of Wingate test protocol with measurement points. WAnT = Wingate anaerobic test, recovery = active recovery at 70 W/100 W. P = Preresting measurement, M1–M8 = measurement points during High-Intensity Interval Training (HIIT) protocol, R3–15 = recovery at minutes 3 to 15. The three different Wingate test protocols were performed in a randomized order regarding the three recovery times (1, 3, or 10 min) with one week recovery in between.

One week after the baseline test, participants completed the first HIIT session. A 30 s WAnT was performed four times, separated by different resting periods (1, 3, or 10 min) (Figure 1). Participants performed the three different HIIT protocols under standardized conditions in a randomized order regarding the three recovery times (1, 3, or 10 min) with one week recovery in between. The power and cadence for the warm-up, cool-down and the active recovery periods was set at 70 W for female and 100 W for male athletes with a cadence of 80–90 rpm. Lactate levels were measured with the enzymatic-amperometric method (Mueller, model Super GL ambulance, Germany) in 10 µL blood taken from the ear lobe. During all tests, gas exchange using a Metalyzer 3B (Cortex, Leipzig, Germany) and heart rate (HR) and beat-to-beat (RR) intervals using a HR monitor (Polar WearLink W.I.N.D-Sender and RS800 CX, Polar Electro GmbH, Büttelborn, Germany) were recorded continuously. For subjective rating of exertion and state of recovery, the Rating of Perceived Exertion (RPE) scale [20] and the Total Quality Recovery scale (TQR) [21] were utilized. RPE was assessed after each of the four WAnT intervals and TQR after each recovery period.

Figure 1 shows the test protocol with the different measurement points. Capillary blood was taken for lactate determination at the first measurement point (P) as well as at the measurement points M2, M4, M6, and M8. Subjective ratings of the perceived exertion (RPE scale) were recorded at M2, M4, M6, and M8. At the measurement points M3, M5, and M7, the subjective rating of the perceived recovery (TQR scale) was recorded. During the recovery period of 10 min, blood was taken for lactate determination at these measurement points as well. Throughout the fifteen-minute cool-down, lactate concentrations were determined at R3, R6, R9, R12, and R15, and the TQR rating was documented. Power, heart rate, and ventilatory parameters were continuously recorded throughout the test period (from P to E15). Intraindividual fatigue, the respective performance decline within the WAnT over the 30-s test duration, was calculated using the formula: %fatigue = (peak powerWAnT − average powerWAnT)/peak powerWAnT × 100).

### 2.3. Statistical Analysis

Statistical analysis was conducted with IBM SPSS Statistics (version 25, International Business Machines Corporation, Armonk, NY, USA) and a published spreadsheet [22]. Descriptive statistics of the data are presented as mean ± standard deviation (SD). Repeated measures two-way ANOVA with Bonferroni corrections for multiple comparisons if warranted was used to detect interaction effects. Where appropriate, univariate post hoc analysis including one-way ANOVA or two-tailed paired t-test were performed with Bonferroni’s correction. The level of *p* < 0.05 was considered statistically significant.

## 3. Results

### 3.1. Power

Participants achieved no significantly different relative peak power outputs—4.69 ± 0.43 W/kg (females) and 5.07 ± 0.50 W/kg (males)—in the baseline testing (*p* = 0.07). Sex differences were smaller when expressed relative to fat-free mass (FFM): 5.57 ± 0.56 W/kg_FFM_ females, 5.63 ± 0.61 W/kg_FFM_ males) (*p* = 0.63) (Table 1).

The mean and standard deviation of Peak Power Output (PP), Average Power Output (AP), and the Percentage Fatigue of the three different Wingate protocols for women and men are displayed in Table 2. Significant differences from WAnT one to WAnT four were found for both PP and AP for women and men in the one-minute recovery protocol. PP also significantly declined from WAnTs one to four for both women and men in the three-minute protocol. However, no significant differences in PP and AP between WAnTs one and four for women and men were found in the three-minute recovery protocol (Table 2). The fatigue (%) decreased from WAnTs one to four in the one-minute and ten-minute recovery protocols for male athletes only.

**Figure 2:**
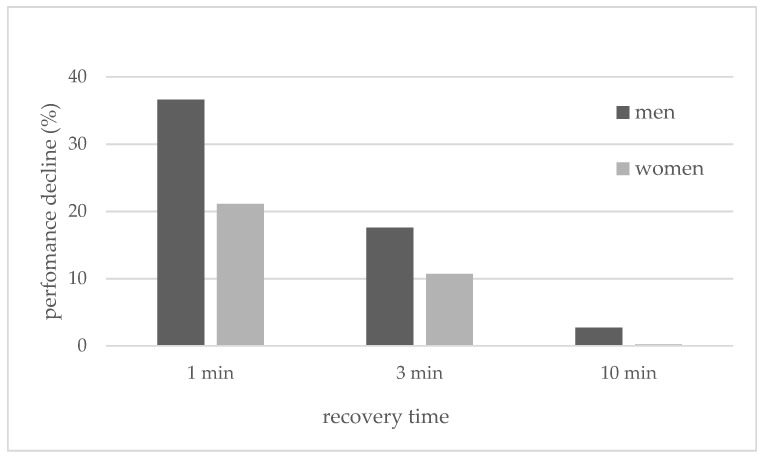
Mean values of the percentage of performance decline (%) during the three different Wingate Test (WAnT) protocols with different recovery periods of one, three and ten minutes for women and men.

The decline in performance (in percentages) between the first and last WAnTs during the different test designs was compared between the sexes using a repeated-measures ANOVA. The descriptive statistics showed that females compared to males consistently had a smaller decrease in performance between the first and last WAnTs (Figure 2).

For both the female and male subjects, the greatest drop in performance was observed in the 1-min recovery and the smallest drop in the 10-min recovery period. The ANOVA analysis did not reveal an interaction effect for %performance drop x sex (F(2, 40) = 0.72, *p* = 0.49). There was also no main effect for the between-subjects factor (F(1, 20) = 2.75, *p* = 0.11). Only a main effect for the within-subject factor was found (F(2, 40) = 55.65, *p* < 0.001).

**Figure 3:**
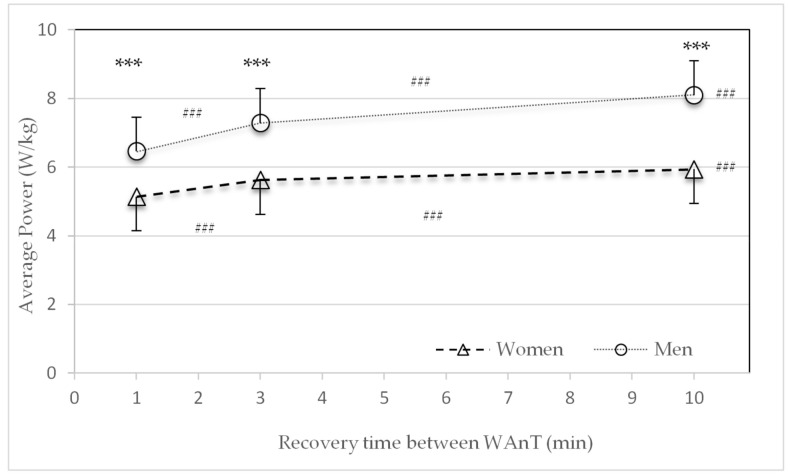
Mean and standard deviation of average power (W/kg) after the second, third, and fourth Wingate tests (WAnTs) in the three different test protocols following the recovery periods of one, three or ten minutes for women and men. *** (*p* < 0.001) between men and women. ^###^ (*p* < 0.001) between the different protocols (1 to 3, 3 to 10, and 1 to 10 min).

**Figure 4:**
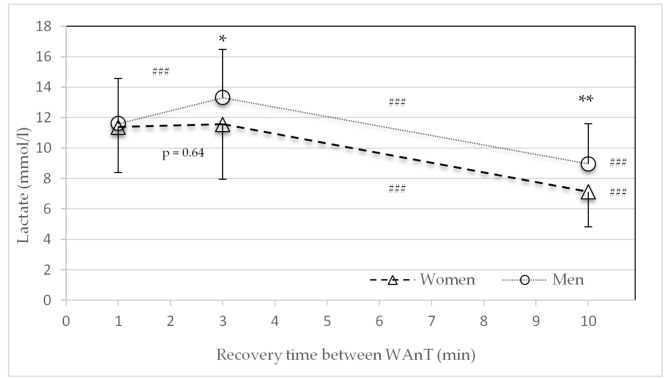
Mean and standard deviation of average lactate concentration (mmol) after the second, third, and fourth Wingate tests (WAnTs) in the three different study protocols following the recovery periods of one, three or ten minutes for women and men. * (*p* < 0.05) and ** (*p* < 0.01) between men and women. ^###^ (*p* < 0.001) between the different protocols (1 to 3, 3 to 10, and 1 to 10 min).

**Figure 5:**
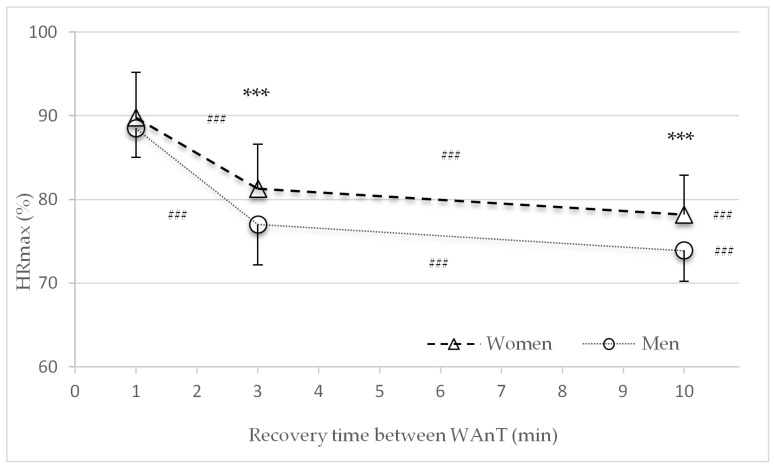
Mean and standard deviation of the average percentage of heart rate recovery (%HRmax) at the end of the second, third, and fourth recovery periods in the three study protocols with different lengths of recovery (one, three, or ten minutes) between Wingate tests (WAnTs) for women and men. *** (*p* < 0.001) between men and women. ^###^ (*p* < 0.001) between the different protocols (1 to 3, 3 to 10, and 1 to 10 min).

**Figure 6:**
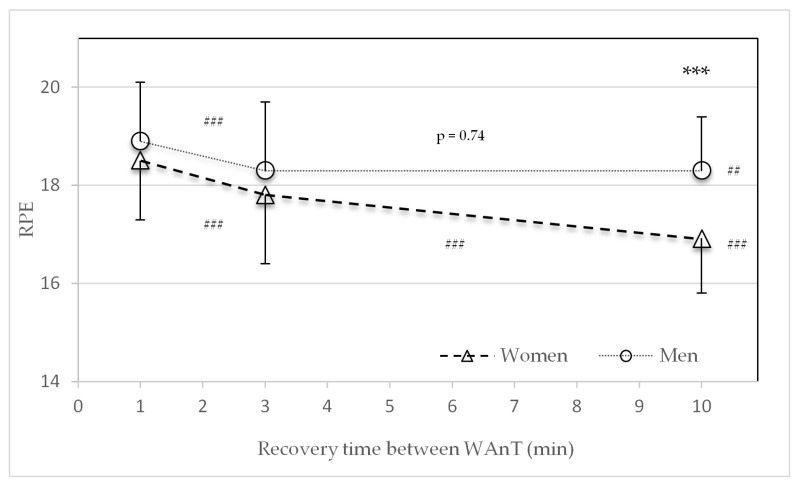
Mean and standard deviation of the average rating of perceived exertion (RPE scale) after the second, third, and fourth Wingate tests (WAnTs) in the three different study protocols with recovery periods of one, three, or ten minutes for women and men. *** (*p* < 0.001) between men and women. ^##^ (*p* < 0.01) and ^###^ (*p* < 0.001) between the different protocols (1 to 3, 3 to 10, and 1 to 10 min).

**Figure 7:**
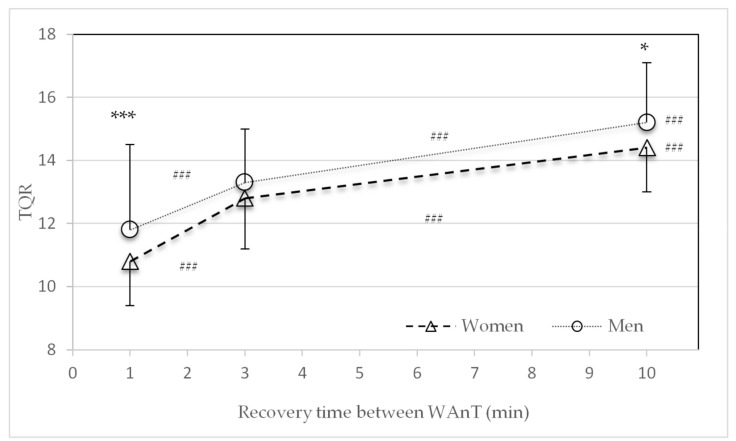
Mean and standard deviation of rating of average perceived recovery (Total Quality Recovery (TQR) scale) at the end of the second, third, and fourth recovery periods in the three different study protocols with different lengths of recovery (one, three, or ten minutes) between Wingate tests (WAnTs) for women and men. * (*p* < 0.05) and *** (*p* < 0.001) between men and women. ^###^ (*p* < 0.001) between the different protocols (1 to 3, 3 to 10, and 1 to 10 min).

Figure 3, Figure 4, Figure 5, Figure 6 and Figure 7 show the data at the measuring points following the second, third, and fourth WAnTs in the three different HIIT protocols with one-, three- or ten-minute active recovery in between the WAnTs.

The relative average power (W/kg) after the second, third and fourth WAnTs was significantly higher in men than in women for all three protocols (*p* < 0.001) (Figure 3). For both sexes the power significantly increased with an increase in recovery time from one to three, three to ten, and one to ten minutes (*p* < 0.001) (Figure 3).

### 3.2. Lactate

There were no significant sex differences in the average lactate concentrations in the HIIT protocol for the one-minute recovery period between the four WAnTs (Figure 4). For the recovery times of three minutes (*p* = 0.04) and ten minutes (*p* = 0.004), men showed significantly higher lactate concentrations. For both sexes, the average lactate concentration significantly declined with an increase in recovery time from three to ten and one to ten minutes (*p* < 0.001). However, the average lactate concentration significantly increased with an increase in recovery time from one to three minutes in men only (*p* < 0.001) (Figure 4).

### 3.3. Heart Rate

Figure 5 shows the percentage of average heart rate recovery (%HR_max_) at the end of the second, third, and fourth recovery periods with different lengths of recovery (one, three, or ten minutes) between WAnTs. No significant sex differences were found in the %HR_max_ in the HIIT protocol with the one-minute recovery period between the four WAnTs. Women had significantly higher %HR_max_ in the recovery times of three minutes (*p* < 0.001) and ten minutes (*p* < 0.001). For both sexes, the %HR_max_ significantly declined with an increase in recovery time from one to three, three to ten and one to ten minutes (*p* < 0.001) (Figure 5).

### 3.4. Subjective Rating of Perceived Exertion (RPE Scale)

There were no significant sex differences in the average RPE values in the HIIT protocol with one- and three-minute recovery periods between the four WAnTs. Regarding the recovery time of ten minutes, only men recorded significantly higher RPE values (*p* > 0.001) (Figure 6). Along with the increase from one to three, three to ten, and one to ten min recovery times, women reported significantly lower average RPE values (*p* < 0.001). Men reported significant lower RPE values from one to three (*p* < 0.001) and one to ten minutes (*p* < 0.01), but not from three to ten minutes (*p* = 0.74) (Figure 6).

### 3.5. Subjective Recovery of Perceived Recovery (TQR Scale)

Significant sex differences in the average TQR values were found for the one-minute (*p* < 0.001) and ten-minute (*p* = 0.03) recovery periods (Figure 7). The average TQR values significantly increased for both women and men with an increase in recovery time from one to three, three to ten, and one to ten minutes (*p* < 0.001).

### 3.6. Ventilatory Parameter during Recovery 

The average respiratory exchange ratio (RER) showed no differences between women and men at all measurement points at the end of the recovery periods for all three HIIT protocols with the different lengths of active recovery periods (1 min recovery: women 1.11 ± 0.08, men 1.12 ± 0.10; 3 min recovery: women 1.02 ± 0.06, men 1.03 ± 0.05; 10 min: women 0.92 ± 0.06, men 0.93 ± 0.06) There was a highly significant decrease in the RER with an increase in the recovery period from one to three and to ten min (*p* < 0.001).

## 4. Discussion

The performance-related parameters VO_2max_ percentile [19] and relative power (W/kg and W/kg_FFM_) attained during the VO_2max_ test were not statistically different between women and men (Table 1). Age was also not statistically different. Differences in body mass, height, and body fat are due to sex-related differences. Thus, it can be postulated that the results were based on two groups with comparable relative aerobic performance levels.

The main findings of this study were that after 30 s of high-intensity all-out cycling exercise, metabolic recovery was faster in women than in men. Significant differences in the lactate concentration were found in the three- and ten-minute recovery time HIIT protocols (Figure 4). These findings are in line with the decline in performance (power) (Figure 2 and Table 2). Compared to men, women consistently showed a smaller decline in average power between the first and last WAnTs. Contrary to this, heart rate and subjective recovery in the active recovery periods between the four WAnTs were slower in women than in men (Figure 5 and Figure 7). Even though women reached a comparable %HR_max_ in the interval bouts, recovery between the interval bouts was slower. This was indicated by a higher %HR_max_ at the end of the three- and ten-minute recovery periods in women. No sex differences in %HR_max_ as well as in lactate concentrations were found in the one-minute recovery period. Women reporting a lower subjective recovery during the recovery periods (Figure 7) might be explained by the reduced heart rate recovery compared to men (Figure 5).

The instructed intensity of the WAnT protocols was an “all-out” effort. Therefore, subjective power input between female and male participants should have always been the same. However, the RPE results showed lower ratings of perceived exertion in the ten-minute recovery protocol for women and an overall decline in RPE with an increase in recovery time for both sexes (Figure 6). Women’s lower RPE values correspond to the lower lactate values in the three- and ten-min protocols. This is in line with Laurent et al. [23] who found no sex differences in RPE and HR_max_ during repeated 30 s sprints. Previous reports [24,25] have shown a higher reliance on fat metabolism during submaximal exercise. The results found in this investigation showed no sex differences in respiratory exchange ratio (RER) during WAnTs.

The present investigation reveals novel information regarding metabolic, cardiovascular, and subjective recovery during 30 s high-intensity intervals (WAnT) with recovery periods of different lengths in well-trained women and men with sex-matched aerobic performance levels (VO_2max_ > 80th percentile (Graves et al. 2015), and relative power). The underlying mechanisms for these findings could be explained by previous studies reporting that women break down 42% less muscle glycogen in type 1 fibers during a single WAnT sprint compared to men [26]. This is in accordance with the findings of lower blood lactate accumulation following single and repeated 30 s sprints [16,23,27]. The reduced glycogenolysis rate may be associated with lower basal activities of muscle phosphofructokinase and lactate dehydrogenase reported in women [28], or a lower catecholamine response to repeated sprints [16,27]. Furthermore, Vincent et al. [29] indicate a sex-related difference in postexercise plasma glucose and insulin responses after a supramaximal exercise.

Esbjörnsson-Liljedahl et al. [26] also showed a significantly higher peak power and average power in males compared to females during a repeated-sprint protocol consisting of repeated WAnTs with 20 min of recovery between tests. Lower lactate levels were reported in women, and a significant decline in average power from sprints one to three was reported in males only. These results, as well as the results by Laurent et al. [22], support the present findings (Table 2 and Figure 2) that females may have a greater ability to restore power between repeated sprints separated by recovery periods and therefore might have a greater maintenance of power compared to men. Additionally, Esbjörnsson-Liljedahl et al. [16] and Laurent et al. [22] found less initial power in female athletes, which is in accordance with the present findings. The greater initial power in men might have led to a greater decline of performance among the different recovery protocols (Figure 2). Overall, performance decline between the four sprints is smaller for women than for men. For both the female and male athletes, the greatest drop in performance is seen in the one-minute recovery design and decreases as the recovery duration increases.

Futhermore, Lievens et al. [30] examined the different responses to high-intensity interval training using WAnTs between a group with a predominance of slow-twitch muscle fibers and another group with a predominance of fast-twitch muscle fibers. Power in the “slow-twitch” group recovered significantly faster than in the “fast-twitch” group. Based on the review by Haizlip et al. [31], who postulated a genetic distribution of slow- and fast-twitch muscle fibers with a higher proportion of slow-twitch fibers in females and a higher proportion of fast-twitch fibers in males, the results of Lievens et al. [30] are in agreement with the present results for the recovery of lactate and power. Results for relative power are shown in W/kg (Figure 3) but comparable findings could have also been shown for relative power expressed as W/kg_FFM_. However, we chose W/kg to reduce limitations in bioimpedance measurements.

Sex differences were also observed to affect the metabolic and sympathetic nervous system responses to supramaximal exercise [27]. Women reported lower plasma catecholamine (adrenaline) and lactate levels 5 min post-WAnTs at similar relative intensities compared to males [27,32]. Thus, this possibly implies an inhibitory effect of oestradiol on the sympathetic nervous system in females [27,32]. Previous research supports the present findings of a slower heart rate recovery in women. While HR_max_ at the end of a maximal running test did not differ between sexes, the decline in heart rate at minute one (HRR1) and minute two (HRR2) was significantly lower in females—i.e., male subjects’ heart rates decreased more rapidly [33]. This is in line with findings by Kappus et al. [34] of HRR1 and HRR2 declining significantly faster in males than in females. Sex differences in autonomic function and vagal reactivation following maximal exercise could explain these findings. Furthermore, Medonca et al. [35] reported that the cardiac autonomic function of women is more affected by supramaximal exercise than that of men.

## 5. Conclusions

As females are under-represented in sports and exercise medicine research, it is therefore not surprising that sex-specific HIIT protocols are widely lacking. HIIT protocols from studies only performed with men are commonly adapted for women. This might be erroneous as the present results and previous studies have indicated that sex-dependent and anthropometric and physiological differences between females and males might significantly affect recovery following repeated high-intensity exercise and thus can affect training response [36,37]. During repeated bouts of exercise women, may be more resistant to fatigue and have a greater ability to recover metabolically, but they have slower heart rates and subjective recovery. As we continue to expand our knowledge on the underlying mechanisms of exercise performance, recovery, and adaptation, we recommend that researchers and practitioners consider the potential sex-specific differences involved.

## Figures and Tables

**Table 1:** Anthropometric data, maximal heart rate (HR_max_), and exercise performance parameters of participants at baseline. Data are means ± SD.

Parameter	Women (n = 11)	Men (n = 11)	*p*-Values
Age (years)	31.7 ± 10.0	33.5 ± 10.2	0.69
Height (m)	1.66 ± 0.06	1.80 ± 0.05	<0.001
Body mass (kg)	57.2 ± 6.3	75.2 ± 4.3	<0.001
BMI (kg/m^2^)	21.2 ± 2.3	23.5 ± 2.2	0.03
Body fat (%)	15.6 ± 6.8	10.9 ± 6.6	<0.001
FFM (kg)	45.9 ± 10.0	67.9 ± 4.0	<0.001
VO_2max_ (mL/min/kg)	47.7 ± 5.8	56.0 ± 5.9	<0.001
HR_max_ (min^−1^)	180.6 ± 11.5	178.9 ± 12.6	0.75
Peak Power (W/kg)	4.69 ± 0.43	5.07 ± 0.50	0.07
Peak Power (W/kg_FFM_)	5.57 ± 0.56	5.63 ± 0.61	0.63
VO_2max_-percentile (%)	91.8 ± 5.5	94.6 ± 5.4	0.23
Training (h/week)	9.6 ± 3.3	8.2 ± 1.9	0.87

**Table 2:** Mean values and standard deviations of peak power output (PP), average power (AP) and %fatigue during Wingate Tests (WAnTs) with one-, three- and ten-min recovery times for women and men. * *p* < 0.0,5, ** *p* < 0.01, *** *p* < 0.001 between T1–T2, T2–T3, and T3–T4.

Parameter	Recovery	Sex	WAnT 1	WAnT 2	WAnT 3	WAnT 4	T1–T4
Peak Power (W)	1 min	w m	532.6 ± 110.9 843.8 ± 174.2	439.2 ± 67.4 *** 673.4 ± 78.7 **	454.8 ± 72.2 625.8 ± 90.7 *	426.8 ± 67.7 573.7 ± 78.7 ***	*p* = 0.006 *p* < 0.001
Av. Power (W)		w m	345.6 ± 54.9 608.0 ± 93.5	302.3 ± 46.2 *** 517.7 ± 69.3 **	292.9 ± 51.7 * 482.5 ± 63.7 ***	287.4 ± 49.5 454.0 ± 66.6 ***	*p* < 0.001 *p* < 0.001
Fatigue (%)		w m	33.6 ± 11.7 27.6 ± 8.0	30.6 ± 10.1 22.9 ± 7.7 *	35.4 ± 7.3 * 22.6 ± 6.1	32.4 ± 8.2 * 20.2 ± 5.9 *	*p* = 0.67 *p* = 0.002
Peak Power (W)	3 min	w m	580.0 ± 98.2 840.5 ± 149.5	555.3 ± 102.7 * 762.8 ± 88.1 *	514.9 ± 97.2 700.7 ± 86.5 ***	505.8 ± 96.1 680.8 ± 83.9	*p* = 0.026 *p* = 0.002
Av. Power (W)		w m	347.9 ± 63.5 622.7 ± 79.7	333.3 ± 53.8 ** 572.4 ± 73.4 ***	319.1 ± 93.5 ** 541.8 ± 70.8 **	313.5 ± 47.2 532.2 ± 71.4 *	*p* = 0.64 *p* = 0.02
Fatigue (%)		w m	39.4 ± 9.3 24.9 ± 9.2	38.9 ± 10.6 24.8 ± 7.3	37.3 ± 8.4 22.6 ± 5.8	37.0 ± 9.6 21.8 ± 5.2	*p* = 0.29 *p* = 0.14
Peak Power (W)	10 min	w m	565.4 ± 99.8 850.6 ± 137.7	522.5 ± 88.2 * 824.5 ± 147.7	517.6 ± 98.2 783.9 ± 124.7	536.9 ± 103.3 781.9 ± 98.0	*p* = 0.06 *p* = 0.09
Av. Power (W)		w m	341.3 ± 59.6 622.1 ± 80.1	340.2 ± 56.5 617.1 ± 81.5	342.9 ± 56.4 603.7 ± 76.9 *	340.1 ± 56.5 605.6 ± 75.1	*p* = 0.80 *p* = 0.25
Fatigue (%)		w m	39.0 ± 9.0 26.1 ± 7.9	34.2 ± 9.1 *** 24.2 ± 9.2	33.0 ± 8.7 22.5 ± 5.1	35.8 ± 8.2 22.3 ± 5.9	*p* = 0.06 *p* = 0.02
